# Circulating thyroid stimulating hormone receptor messenger RNA and differentiated thyroid cancer: A diagnostic meta-analysis

**DOI:** 10.18632/oncotarget.14251

**Published:** 2016-12-27

**Authors:** Zhen-Zhen Zhang, Qiang Chen, Chao-Yue Kong, Zhan-Ming Li, Li-Shun Wang

**Affiliations:** ^1^ Institute of Biomedical Sciences, Minhang Hospital, Fudan University, Shanghai, P.R. China; ^2^ School of Public Health Taishan Medical University, Shandong, P.R. China

**Keywords:** thyroid stimulating hormone receptor, thyroid cancer, diagnostic meta-analysis

## Abstract

Thyroid stimulating hormone receptor messenger RNA (TSHR-mRNA) is over-expressed in thyroid cancer patients, which indicates that TSHR-mRNA is a potential biomarker of thyroid cancer. However, system evaluation for TSHR-mRNA as a diagnostic biomarker of thyroid cancer is deficient. The performance of TSHR-mRNA for thyroid cancer diagnosis was evaluated in this study. Three common international databases as well as a Chinese database were applied for literature researching. Quality assessment of the included literatures was conducted by the QUADAS-2 tool. Totally, 1027 patients from nine studies eligible for the meta-analysis were included in this study. Global sensitivity and specificity for the positivity of TSHR-mRNA in the thyroid cancer diagnosis is 72% and 82%. The value of AUC for this test performance was 0.84. Our meta-analysis suggests that TSHR-mRNA might be a potential biomarker to complete present diagnostic methods for early and precision diagnosis of thyroid cancer. Notably, this findings need validation thorough large-scale clinical studies.

## INTRODUCTION

Thyroid cancer is one of the most common cancers in the endocrine system, and the incidence of thyroid cancer is about 1%, and it is also the main cause of death in the endocrine system [1]. There are around 44670 new cases and 1690 deaths of thyroid cancer every year [2]. In the past years, the average annual percentage for thyroid cancer increased 5.3% per year [3]. Currently, the diagnostic methods of thyroid cancer mainly include ultrasound, CT and fine-needle aspiration cytology. In a certain extent, it can improve the accuracy of diagnosis of thyroid cancer. However, surveillance, early and precision diagnosis as well as treatment are still the main problem [4]. Currently there are ThyroSeqV2 next generation sequencing assay to improve the diagnosis of benign and malignant thyroid cancer method [5], which is high accuracy but high cost, and can not be popularized. So new methods is necessary in the diagnosis of thyroid cancer.

Thyroid stimulating hormone receptor (TSHR) is a specific protein of thyroid cells, which is found on the membrane of thyroid gland cells. It plays an important role in the development of thyroid function and thyroid disease. Because TSHR-mRNA is over-expressed in thyroid cancer cells, the measurement of TSHR-mRNA in the circulation is very valuable for the diagnosis of thyroid cancer [4]. In recent years, there have been many reports of TSHR-mRNA in the diagnosis of thyroid cancer. However, a system evaluation of TSHR-mRNA as a diagnostic biomarker for thyroid cancer is deficient, so we made this first diagnosis meta-analysis of TSHR-mRNA on thyroid cancer.

## RESULTS

### Characteristics and quality of the included studies

**Figure 1:**
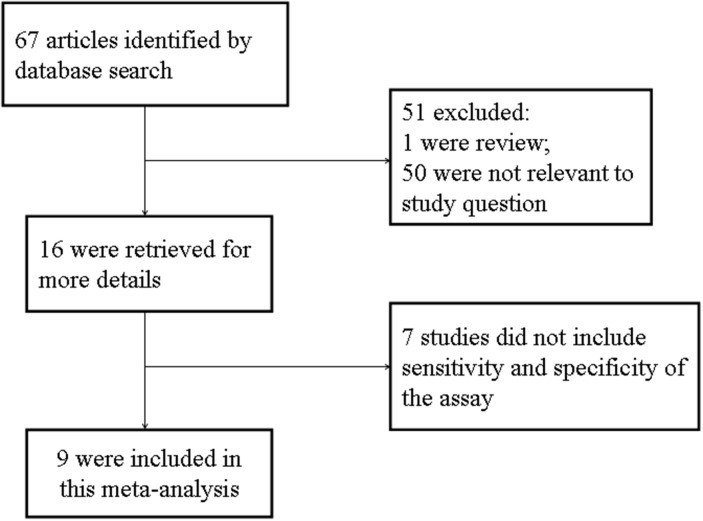
Flow chart of selection process for eligible studies

The literature search identified 67 relevant articles. Titles and abstracts were preliminarily reviewed and a total of 55 articles were excluded for various reasons (including literature type is review, case reports and letter, or studies do not solely focus on TSHR-mRNA and/or not specifically pertain to thyroid cancer). The article selection process is summarized in Figure 1. Finally, nine publications were included for this meta-analysis [6–14].

The nine included studies were published between 2005 and 2015, of which 1027 patients were involved. 4 out of 9 articles were published in China. RT-PCR was applied for TSHR-mRNA detection, with histopathological examination as the gold standard for thyroid cancer diagnosis. The detailed characteristics of these studies are listed in Table 1.

**Table 1:** Main characters of the studies included in the meta-analysis

Studies	year	country	patients	Reference standard	Patient type	cut-off(ng/ug)	TP	FP	FN	TN
Wagner, K	2005	USA	46	FNA and/OR Surgical pathology	All TC^a^; FNA indeterminate^b^	1.02	16	5	6	19
Chia, S.Y	2007	USA	131	FNA and/OR Surgical pathology	All TC	1.02	56	15	21	39
Milas, M	2010	USA	368	Surgical pathology	All TC	1	124	29	78	137
Salwa, H	2011	Egypt	40	FNA	All TC; FNA indeterminate	1.02	20	2	4	14
Gutnick, J	2012	USA	39	FNA and/OR Surgical pathology	All TC	1	8	5	3	23
Jiang, LH	2012	China	126	Surgical pathology	All TC	1.02	56	1	0	69
Liu, X W	2012	China	130	FNA and/OR Surgical pathology	All TC	1.02	26	11	24	69
Ren, T T	2013	China	67	FNA and/OR Surgical pathology	All TC	1.02	31	16	3	17
Jiang, W	2015	China	80	Surgical pathology	All TC	1.5	45	6	7	22

TP (true-positive), FP (false-positive), FN (false-negative), TN (true-negative), c-c(case-control), cut-off(Positive boundary value).

a : All thyroid cancer.

b : FNA diagnosis of indeterminate.

**Figure 2:**
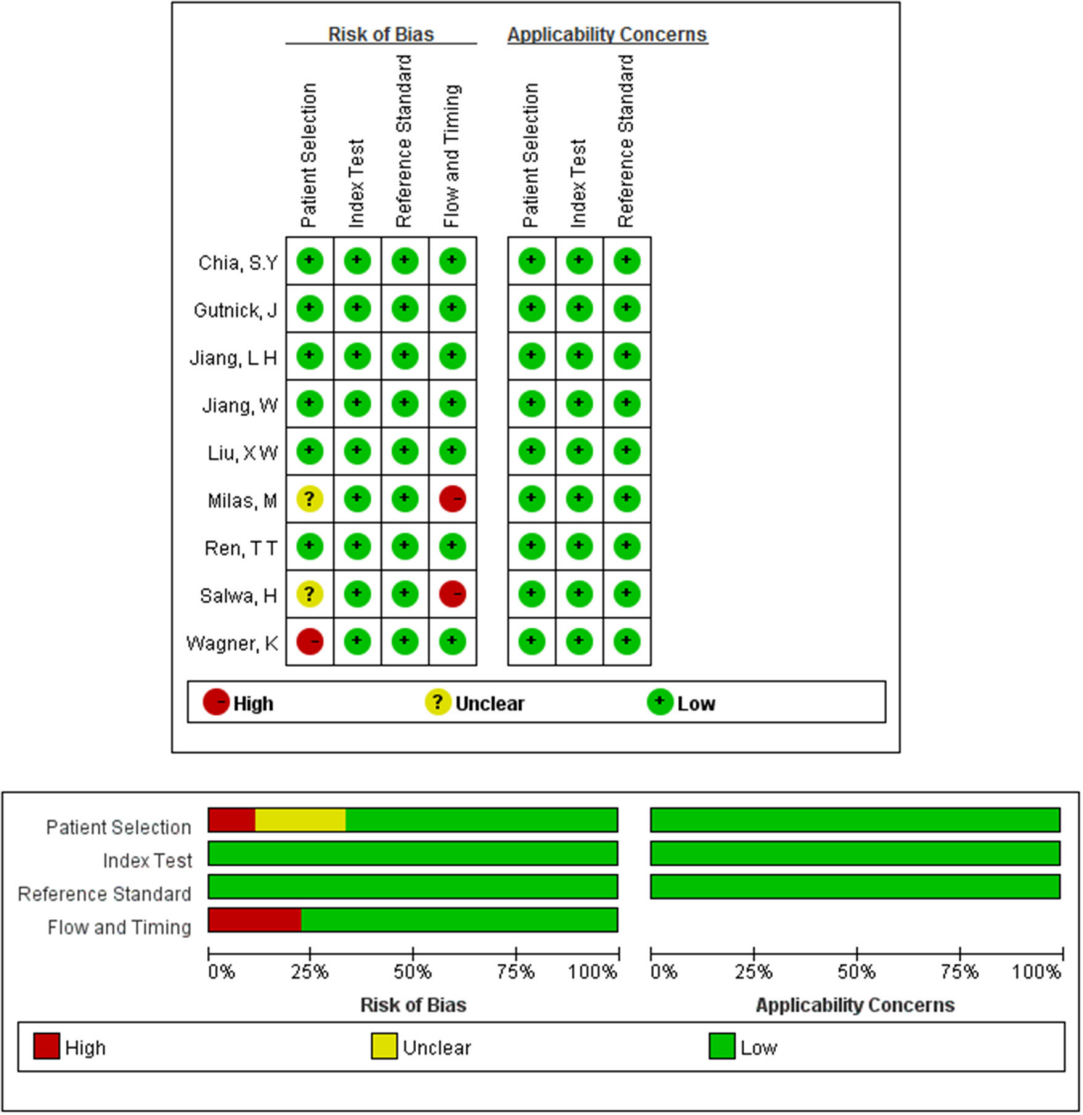
Quality assessments of included studies by using the QUADAS-2 tool Methodological quality was generally better. The risk of bias in terms of flow and timing and patient selection and was met by less than 30% of the studies. Only three studies unfulfilled all criteria.

The methodological quality of the studies according to the QUADAS-2 tool is summarized in Figure 2, and the methodological quality was generally better.

### Overall analysis

**Figure 3:**
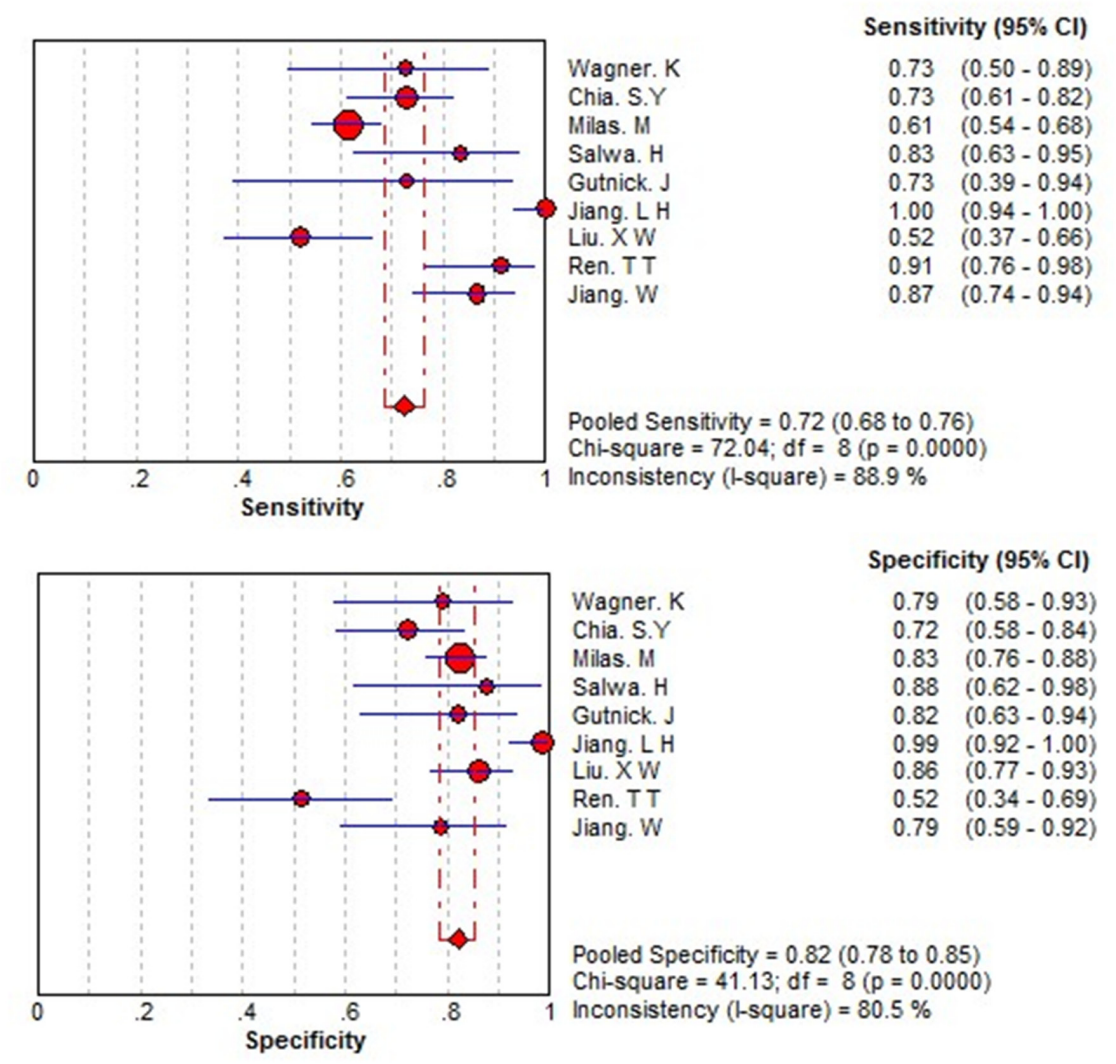
Forest plots of the sensitivity and specificity for THSR-mRNA in the diagnosis of thyroid cancer The point estimates of sensitivity and specificity for each study are shown as solid circles and size of each solid circle indicates the sample size of each study. Error bars are 95% confidence intervals.

**Figure 4:**
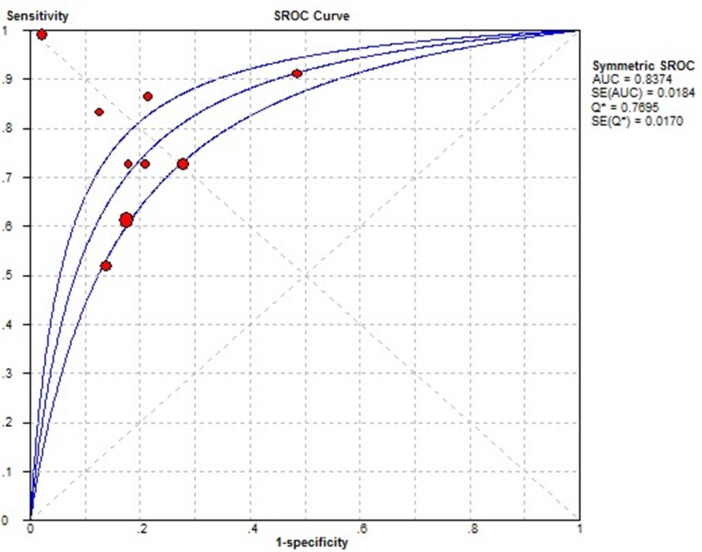
Summary receive operating characteristic (SROC) curve for THSR-mRNA in the diagnosis of thyroid cancer Solid circles represent each study included in the meta-analysis. The size of each solid circle indicates the size of each study. The regression SROC curve summarizes the overall diagnostic accuracy.

There was heterogeneity presented in this meta-analysis, as revealed by the results (Q = 21.979, p =0.000, I^2^=91). Thus, a random effects model was used for the further analysis. As shown in Figure 3, the sensitivity and specificity for the pooled data were 0.72 (0.68 to 0.76) and 0.82 (0.78 to 0.85), respectively. The AUC was 0.84 (Figure 4), and the DOR was 12.93 (6.89 to 24.24). The results corresponded to a PLR (Positive Likelihood Ratio) of 3.71 (2.51, 5.50) and an NLR (Negative Likelihood Ratio) of 0.31 (0.21, 0.46). High heterogeneity was demonstrated in the sensitivity (I^2^= 88.9%) and specificity (I^2^ = 80.5%).

### Threshold effect and publication bias

To retrospect the cause of high heterogeneity, the threshold was firstly analyzed. There was no threshold effect presenting in this meta-analysis, which was supported by the spearman correlation coefficient [15] value of 0.100 (p = 0.862; p > 0.05).

Besides threshold effect, heterogeneity can also be contributed by socio-demographic characteristics and geographical location [16]. Then, geographical location and patient characters were analyzed with a meta-regression. No significant heterogeneity was demonstrated in terms of geographical location (coefficient = 0.220, p = 0.283) and patient characters (coefficient = 0.000, p = 0.988). Therefore, other factors might contribute to the observed high heterogeneity.

**Figure 5:**
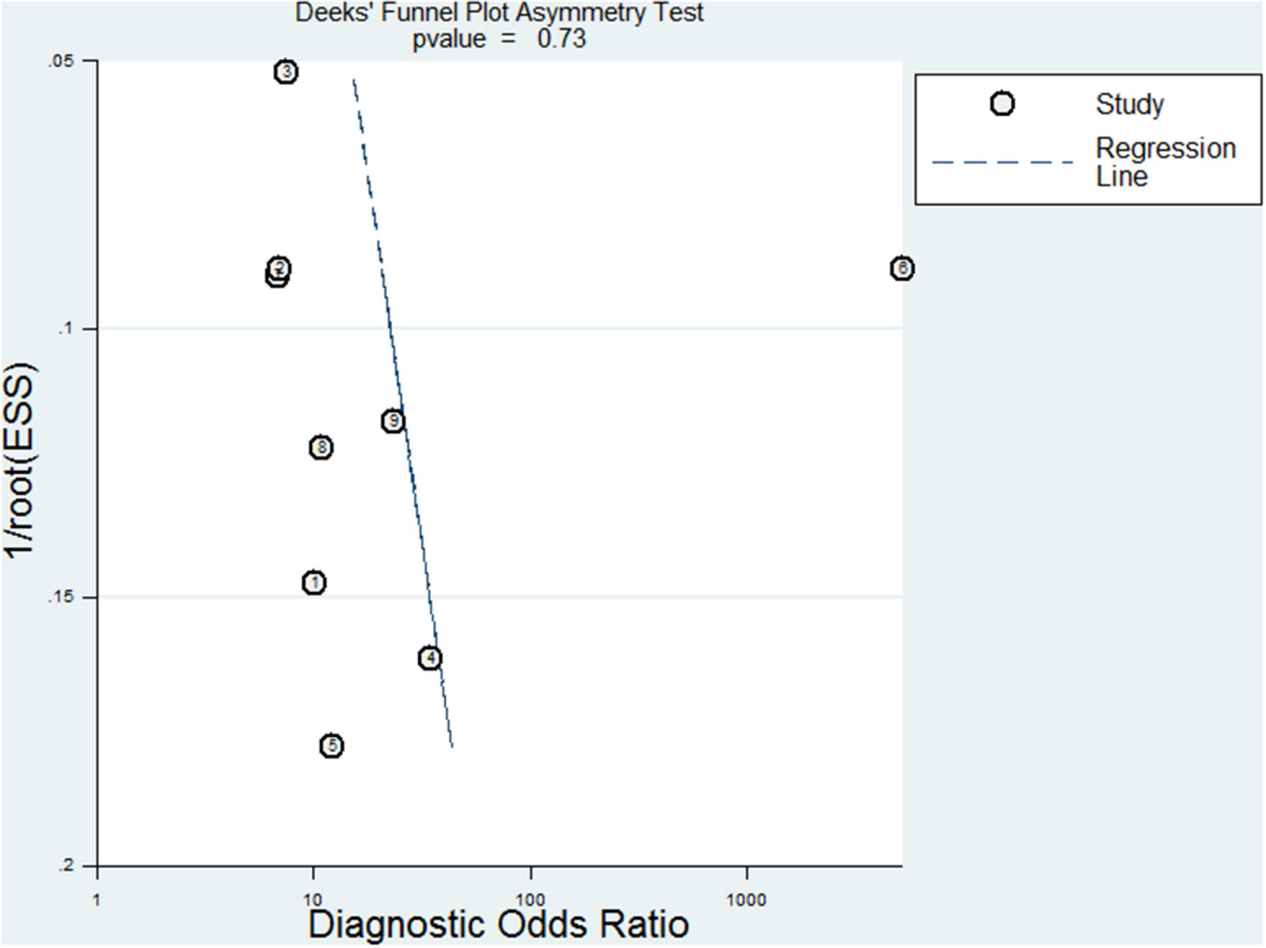
Deek's plots for the assessment of publication bias The funnel graph plots the log of the diagnostic odds ratio (DOR) against the standard error of the log of the DOR (an indicator of sample size). Solid circles represent each study in the meta-analysis. The line indicates the regression line.

Furthermore, the potential publication bias was evaluated by Deeks’ funnel plots. The p-value of 0.73 suggested there is no publication bias (Figure 5).

### Sensitivity analysis

The sensitivity value range is 0.69 (0.65-0.73) to 0.79 (0.74-0.83), specificity value range of 0.79 (0.75-0.83) to 0.84 (0.81-0.87) the AUC of the range is 0.829 to 0.884. Sensitivity analysis showed that the results were stable and there was no bias.

## DISCUSSION

The preoperative evaluation of thyroid nodules currently depends on fine needle aspiration (FNA) biopsy. However, FNA suffer inherent limitations. Its inadequate sampling may occasionally led to cancer cell missing and 15–30% of cases are indeterminate [17–19]. In addition, FNA is virtually impossible to differentiate follicular cancers (FC) from benign follicular adenomas (FA), and thus molecular markers is urgently necessary for this purpose [20–24].

Thyroid and thyroid cancer cells specifically express TSHR-mRNA, increasing evidences have demonstrated that circulating TSHR-mRNA is involved in cancer diagnosis and prognosis. The latest article shows that TSHR-mRNA is a molecular marker of thyroid cancer cells circulating [25]. However, there is no systematic evaluation for these studies.

In this work, we conducted the first diagnostic meta-analysis to draw a system evaluation for TSHR-mRNA on the diagnosis of thyroid cancer. An AUC of 0.8427 showed that circulating TSHR-mRNA may be a promising biomarker to discriminate thyroid cancer patients from benign thyroid nodules individuals, with a summary sensitivity of 72% and specificity of 83%. As a potential diagnostic biomarker for thyroid cancer, serum or plasma TSHR-mRNA is characterized by minimal invasion and convenience compared with FNA based histopathological examination. In addition, TSHR-mRNA could serve to monitor the disease recurrence after surgery [26].

However, as a novel biomarker, circulating TSHR-mRNA for thyroid cancer diagnosis have been conducted in limited studies [27]. Therefore, no large number of patient can be included in this meta-analysis. The finding in this work need validation in large-scale clinical studies. Further more, we supposed TSHR-mRNA combined with FNA should have better performance than anyone alone [13, 28–30]. Unfortunately, these studies were too few for further analysis.

Overall, this meta-analysis indicates that circulating TSHR-mRNA is potential to be a biomarker for early-stage thyroid cancer diagnosis. However, future clinical studies are necessary to validate this finding.

## MATERIALS AND METHODS

### Search strategy and study selection

The method of literature search is the same as we have just published [31, 32]. With the Medical Subject Headings or text words ‘TSHR mRNA’, combined with ‘Thyroid cancer’ or ‘Thyroid neoplasm’, a computerized search were conducted for studies that were published between 1972 and February 2015 in the databases of PUBMED, MEDLINE and EMBASE and Chinese National Knowledge Infrastructure. Moreover, the reference lists of retrieved articles were manually searched for additional relevant studies. No language restrictions were applied.

Studies that is inability to obtain individual data, review articles, case reports, using the same sample, absence of case or control groups, using specimens other than human, using specimens other than those obtained exclusively from the blood (for example, thyroid gland tissue and derivatives) were excluded.

### Data extraction and quality assessment

The data were independently collected from the included studies and reached a consensus on all items by two researchers. Any disagreements would be resolved by discussion with a third author for a final consensus. The following information was collected: reference, number of patients, technique employed, histological types of neoplasias. In addition, the following data were extracted: first author's name, publication year, country, cutoff value, patients size, TP (true-positive), FP (false-positive), FN (false-negative) and TN (true-negative).

Methodological quality of the primary studies was assessed by the Quality Assessment of Diagnostic Accuracy Studies 2 (QUADAS-2) checklist [33].

### Meta analysis

Statistical analysis was conducted with Meta-DiSc 1.4 and Stata 12.0 [15]. In the analysis, benign thyroid cancer served as negative control. P < 0.05 was set as statistical significance. A value of I^2^ > 50% and/or a p < 0.05 indicated the between-study heterogeneity [34]. When heterogeneity was presented in the study, a random effects model was applied to sensitivity, specificity, likelihood ratio, and diagnostic odds ratio. The threshold effect was investigated based on the spearman correlation coefficient. A summary receiver operating characteristic curve (SROC) was used for the overall evaluation of diagnostic performance. Publication bias was assessed using Deeks’ funnel plot analysis [35].

## References

[1] Are C, Shaha AR (2006). Anaplastic thyroid carcinoma: biology, pathogenesis, prognostic factors, and treatment approaches. Annals of surgical oncology.

[2] Jemal A, Siegel R, Xu J, Ward E (2010). Cancer statistics, 2010. CA Cancer J Clin.

[3] Magreni A, Bann DV, Schubart JR, Goldenberg D (2015). The effects of race and ethnicity on thyroid cancer incidence. JAMA Otolaryngol Head Neck Surg.

[4] Barbosa GF, Milas M (2008). Peripheral thyrotropin receptor mRNA as a novel marker for differentiated thyroid cancer diagnosis and surveillance. Expert Rev Anticancer Ther.

[5] Nikiforov YE, Carty SE, Chiosea SI, Coyne C, Duvvuri U, Ferris RL, Gooding WE, Hodak SP, LeBeau SO, Ohori NP, Seethala RR, Tublin ME, Yip L, Nikiforova MN (2014). Highly accurate diagnosis of cancer in thyroid nodules with follicular neoplasm/suspicious for a follicular neoplasm cytology by ThyroSeq v2 next-generation sequencing assay. Cancer.

[6] Chia SY, Milas M, Reddy SK, Siperstein A, Skugor M, Brainard J, Gupta MK (2007). Thyroid-stimulating hormone receptor messenger ribonucleic acid measurement in blood as a marker for circulating thyroid cancer cells and its role in the preoperative diagnosis of thyroid cancer. J Clin Endocrinol Metab.

[7] Gutnick J, Soldes O, Gupta M, Milas M (2012). Circulating thyrotropin receptor messenger RNA for evaluation of thyroid nodules and surveillance of thyroid cancer in children. Journal of pediatric surgery.

[8] Milas M, Shin J, Gupta M, Novosel T, Nasr C, Brainard J, Mitchell J, Berber E, Siperstein A (2010). Circulating thyrotropin receptor mRNA as a novel marker of thyroid cancer: clinical applications learned from 1758 samples. Ann Surg.

[9] Pak K, Cheon GJ, Kang KW, Kim SJ, Kim IJ, Kim EE, Lee DS, Chung JK (2014). The effectiveness of recombinant human thyroid-stimulating hormone versus thyroid hormone withdrawal prior to radioiodine remnant ablation in thyroid cancer: a meta-analysis of randomized controlled trials. Journal of Korean medical science.

[10] Plotkin M, Hautzel H, Krause BJ, Schmidt D, Larisch R, Mottaghy FM, Boemer AR, Herzog H, Vosberg H, Muller-Gartner HW (2002). Implication of 2-18fluor-2-deoxyglucose positron emission tomography in the follow-up of Hurthle cell thyroid cancer. Thyroid.

[11] Stokkel MP, Duchateau CS, Dragoiescu C (2006). The value of FDG-PET in the follow-up of differentiated thyroid cancer: a review of the literature. The quarterly journal of nuclear medicine and molecular imaging.

[12] Tu J, Wang S, Huo Z, Lin Y, Li X, Wang S (2014). Recombinant human thyrotropin-aided versus thyroid hormone withdrawal-aided radioiodine treatment for differentiated thyroid cancer after total thyroidectomy: a meta-analysis. Radiotherapy and oncology.

[13] Wagner K, Arciaga R, Siperstein A, Milas M, Warshawsky I, Sethu S, Reddy K, Gupta MK (2005). Thyrotropin receptor/thyroglobulin messenger ribonucleic acid in peripheral blood and fine-needle aspiration cytology: diagnostic synergy for detecting thyroid cancer. J Clin Endocrinol Metab.

[14] Webb RC, Howard RS, Stojadinovic A, Gaitonde DY, Wallace MK, Ahmed J, Burch HB (2012). The utility of serum thyroglobulin measurement at the time of remnant ablation for predicting disease-free status in patients with differentiated thyroid cancer: a meta-analysis involving 3947 patients. The Journal of clinical endocrinology and metabolism.

[15] Zamora J, Abraira V, Muriel A, Khan K, Coomarasamy A (2006). Meta-DiSc: a software for meta-analysis of test accuracy data. BMC Med Res Methodol.

[16] Lijmer JG, Bossuyt PM, Heisterkamp SH (2002). Exploring sources of heterogeneity in systematic reviews of diagnostic tests. Statistics in medicine.

[17] Caruso G, Tabarri B, Lucchi I, Tison V (1990). Fine needle aspiration cytology in a case of diffuse sclerosing carcinoma of the thyroid. Acta cytologica.

[18] Castro MR, Gharib H (2005). Continuing controversies in the management of thyroid nodules. Annals of internal medicine.

[19] Alexander EK, Kennedy GC, Baloch ZW, Cibas ES, Chudova D, Diggans J, Friedman L, Kloos RT, LiVolsi VA, Mandel SJ, Raab SS, Rosai J, Steward DL, Walsh PS, Wilde JI, Zeiger MA (2012). Preoperative diagnosis of benign thyroid nodules with indeterminate cytology. N Engl J Med.

[20] Barden CB, Shister KW, Zhu B, Guiter G, Greenblatt DY, Zeiger MA, Fahey TJ (2003). Classification of follicular thyroid tumors by molecular signature: results of gene profiling. Clinical cancer research.

[21] Cerutti JM, Delcelo R, Amadei MJ, Nakabashi C, Maciel RM, Peterson B, Shoemaker J, Riggins GJ (2004). A preoperative diagnostic test that distinguishes benign from malignant thyroid carcinoma based on gene expression. The Journal of clinical investigation.

[22] Rosen J, He M, Umbricht C, Alexander HR, Dackiw AP, Zeiger MA, Libutti SK (2005). A six-gene model for differentiating benign from malignant thyroid tumors on the basis of gene expression. Surgery.

[23] Weber F, Eng C (2005). Gene-expression profiling in differentiated thyroid cancer--a viable strategy for the practice of genomic medicine?. Future oncology.

[24] Zeiger MA, Dackiw AP (2005). Follicular thyroid lesions, elements that affect both diagnosis and prognosis. Journal of surgical oncology.

[25] Aliyev A, Gupta M, Nasr C, Hatipoglu B, Milas M, Siperstein A, Berber E (2015). Circulating Thyroid-Stimulating Hormone Receptor Messenger Rna as a Marker of Tumor Aggressiveness in Patients with Papillary Thyroid Microcarcinoma. Endocr Pract.

[26] Milas M, Barbosa GF, Mitchell J, Berber E, Siperstein A, Gupta M (2009). Effectiveness of peripheral thyrotropin receptor mRNA in follow-up of differentiated thyroid cancer. Ann Surg Oncol.

[27] Hoang-Vu C, Dralle H, Scheumann G, Maenhaut C, Horn R, von zur Muhlen A, Brabant G (1992). Gene expression of differentiation- and dedifferentiation markers in normal and malignant human thyroid tissues. Exp Clin Endocrinol.

[28] Caraway NP, Sneige N, Samaan NA (1993). Diagnostic pitfalls in thyroid fine-needle aspiration: a review of 394 cases. Diagnostic cytopathology.

[29] Gharib H (1994). Fine-needle aspiration biopsy of thyroid nodules: advantages, limitations, and effect. Mayo Clinic proceedings.

[30] Aliyev A, Patel J, Brainard J, Gupta M, Nasr C, Hatipoglu B, Siperstein A, Berber E (2016). Diagnostic accuracy of circulating thyrotropin receptor messenger RNA combined with neck ultrasonography in patients with Bethesda III-V thyroid cytology. Surgery.

[31] Li ZM, Wu ZX, Han B, Mao YQ, Chen HL, Han SF, Xia JL, Wang LS (2016). The association between BMI and gallbladder cancer risk: a meta-analysis. Oncotarget.

[32] Hu N, Li ZM, Liu JF, Zhang ZZ, Wang LS (2016). An overall and dose-response meta-analysis for thyrotropin and thyroid cancer risk by histological type. Oncotarget.

[33] Whiting PF, Rutjes AW, Westwood ME, Mallett S, Deeks JJ, Reitsma JB, Leeflang MM, Sterne JA, Bossuyt PM, Group Q- (2011). QUADAS-2: a revised tool for the quality assessment of diagnostic accuracy studies. Annals of internal medicine.

[34] Huedo-Medina TB, Sanchez-Meca J, Marin-Martinez F, Botella J (2006). Assessing heterogeneity in meta-analysis: Q statistic or I2 index?. Psychol Methods.

[35] Deeks JJ, Macaskill P, Irwig L (2005). The performance of tests of publication bias and other sample size effects in systematic reviews of diagnostic test accuracy was assessed. J Clin Epidemiol.

